# K13 mutations and *pfmdr1* copy number variation in *Plasmodium falciparum* malaria in Myanmar

**DOI:** 10.1186/s12936-016-1147-3

**Published:** 2016-02-24

**Authors:** Aye A. Win, Mallika Imwong, Myat P. Kyaw, Charles J. Woodrow, Kesinee Chotivanich, Borimas Hanboonkunupakarn, Sasithon Pukrittayakamee

**Affiliations:** Department of Medicine, Institute of Medicine 1, Yangon, Myanmar; Department of Molecular Tropical Medicine and Genetics, Mahidol University, Bangkok, Thailand; Mahidol Oxford Tropical Medicine Research Unit, Bangkok, Thailand; Department of Medical Research (Lower Myanmar), Yangon, Myanmar; Nuffield Department of Clinical Medicine, Centre for Tropical Medicine & Global Health, University of Oxford, Oxford, UK; Faculty of Tropical Medicine, Mahidol University, Bangkok, Thailand

**Keywords:** Malaria, *Plasmodium falciparum*, K13 mutation, *pfmdr1* copy number variation, Artemisinin resistance, Myanmar

## Abstract

**Background:**

Artemisinin-based combination therapy has been first-line treatment for falciparum malaria in Myanmar since 2005. The wide extent of artemisinin resistance in the Greater Mekong sub-region and the presence of mefloquine resistance at the Myanmar-Thailand border raise concerns over resistance patterns in Myanmar. The availability of molecular markers for resistance to both drugs enables assessment even in remote malaria-endemic areas.

**Methods:**

A total of 250 dried blood spot samples collected from patients with *Plasmodium falciparum* malarial infection in five malaria-endemic areas across Myanmar were analysed for *kelch 13* sequence (*k13*) and *pfmdr1* copy number variation. *K13* mutations in the region corresponding to amino acids 210–726 (including the propeller region of the protein) were detected by nested PCR amplification and sequencing, and *pfmdr1* copy number variation by real-time PCR. In two sites, a sub-set of patients were prospectively followed up for assessment of day-3 parasite clearance rates after a standard course of artemether-lumefantrine.

**Results:**

*K13* mutations and *pfmdr1* amplification were successfully analysed in 206 and 218 samples, respectively. Sixty-nine isolates (33.5 %) had mutations within the *k13* propeller region with 53 of these (76.8 %) having mutations already known to be associated with artemisinin resistance. F446I (32 isolates) and P574L (15 isolates) were the most common examples. *K13* mutation was less common in sites in western border regions (29 of 155 isolates) compared to samples from the east and north (40 of 51 isolates; p < 0.0001). The overall proportion of parasites with multiple *pfmdr1* copies (greater than 1.5) was 5.5 %. Seven samples showed both *k13* mutation and multiple copies of *pfmdr1*. Only one of 36 patients followed up after artemether-lumefantrine treatment still had parasites at day 3; molecular analysis indicated wild-type *k13* and single copy *pfmdr1*.

**Conclusion:**

The proportion of *P. falciparum* isolates with mutations in the propeller region of *k13* indicates that artemisinin resistance extends across much of Myanmar. There is a low prevalence of parasites with multiple *pfmdr1* copies across the country. The efficacy of artemisinin-based combination therapy containing mefloquine and lumefantrine is, therefore, expected to be high, although regular monitoring of efficacy will be important.

**Electronic supplementary material:**

The online version of this article (doi:10.1186/s12936-016-1147-3) contains supplementary material, which is available to authorized users.

## Background

The morbidity and mortality of falciparum malaria have reduced remarkably in many malaria-endemic areas following the expanding use of insecticide-treated bed nets and artemisinin-based combination therapy (ACT) [1], although more than half a million deaths still occur annually [2]. The emergence of resistance to ACT in Cambodia [3, 4] and Thailand [5, 6] threatens to undermine further reductions in malaria mortality, and tracking the extent of anti-malarial resistance is urgent given the consequences of the previous global spread of chloroquine and sulfadoxine-pyrimethamine resistance.

Myanmar is likely to become a critical site for controlling malaria parasites and malaria remains one of its diseases of national concern. In 2013, malarial morbidity and mortality rates were 6.44/1000 population and 0.48/100,000 population, respectively, a considerable decrease in disease burden when compared to 1990, when morbidity and mortality were 24.4/1000 and 12.6/100,000, respectively. Three ACT formulations (artemether-lumefantrine, artesunate-mefloquine and dihydroartemisinin-piperaquine) have been deployed as first-line treatment for acute uncomplicated *Plasmodium falciparum* malaria since 2005, with artemether-lumefantrine generally used in Ministry of Health facilities and dihydroartemisinin (DHA)-piperaquine used in military facilities. However, artemisinin monotherapy remains in use in the private sector [7]. There is currently no evidence of unsatisfactory efficacy of any of these ACT within Myanmar, although few therapeutic efficacy studies have been undertaken [8, 9]. Reduced in vivo responses to artemisinins have been confirmed in the southern extremity of Myanmar [10] and at the Myanmar-Thai [5, 6, 11] and Myanmar-China borders [12].

The primary underlying molecular factors in resistance to several anti-malarials are known, allowing molecular surveillance of resistance in remote or logistically challenging environments where in vivo and in vitro studies are impractical. Artemisinin resistance is determined by mutation in the propeller region of the *P. falciparumkelch protein* gene on chromosome 13 (*k13*) [13–15] while mefloquine and lumefantrine resistance results from increased *pfmdr1* copy number [16].

The main objective of the current study was to track the likely efficacy of artemether-lumefantrine and other ACT in several regions in Myanmar using the *k13* and *pfmdr1* copy number molecular markers.

## Methods

### Screening of patients and sample collection

Dried blood spot samples were collected from patients with microscopically confirmed *P. falciparum* infection during a one-year period from October 2013 to September 2014 in five malaria-endemic areas. The studied sites were Ann in Rakhine State, Homemalin in Sagaing State, Myit Kyi Nar in Kachin State, Kyauk Me in Northern Shan State, and Phruso in Kayah State. Accurate and recent *P. falciparum* transmission data are not available from these sites, although up to 2010 the three administrative regions spanning the western Myanmar border (Rakhine and Chin States and Sagaing Region) appeared to have the highest transmission levels in the country (generally PfPR2-10 >5 to <40 %, or ‘intermediate levels’) with lower prevalence in central and eastern areas [17].

Febrile patients in the respective study sites were recruited by passive case sampling at local clinics run by the Vector Borne Disease Control Programme and by active case sampling at nearby villages. Positive rapid diagnostic test (RDT) results for falciparum malaria were confirmed by thick and thin blood film examination. Patients with parasite counts of 250 parasites/µl or more in the thick blood film were eligible for the study. After obtaining written informed consent, dried blood spot samples from participants were collected on Whatman 3MM filter paper (Maidstone, UK). The short-term efficacy of artemether-lumefantrine therapy via assessment of day-3 parasitaemia (after completion of directly observed treatment) in a sub-set of patients studied in Homemalin and Kyauk Me were also done. The study was approved by the Ethical Committees of Faculty of Tropical Medicine, Mahidol University and Department of Medical Research (lower Myanmar).

### Detection of *k13* mutation

*K13* sequences corresponding to amino acids 210–726 were obtained; results from an initial set of 74 samples were published as a part of a previous study [18] (Additional file 1). Nested PCR methods were as described previously [14]. Briefly, each PCR reaction contained 10 mM Tris-HCL buffer, 2 mM or 3 mM MgCl_2_, 250 µM 4-deoxynucleotide triphosphate (dNTPs), 250 nM oligonucleotide primers, 0.5 units (2 units for the second nested PCR) Platinum Taq DNA polymerase (Invitrogen, USA) and 2 µL of genomic DNA template (or 5 µL of PCR product from the first nested PCR) within a final reaction volume of 100 µL. A total of 25 PCR cycles for nested 1 and 35 cycles for nested 2 were undertaken using a MyCycler ™ thermal cycler (Bio-Rad Laboratories, USA). The temperature cycles consisted of pre-denaturation at 94 °C for 5 min followed by 94 °C for 1 min, 58 °C for 2 min (1 min for the second nested PCR), 72 °C for 2 min (1 min for nested 2) and final extension at 72 °C for 10 min. DNA template from the AQ strain (without *k13* mutation) was used as a positive control and a DNA free template was also included as negative control in each PCR reaction. Sequencing of *k13* was obtained at Macrogen, Republic of Korea. Multiple nucleotide sequence alignments were analysed by BioEdit version 7.1.3.0, using 3D7 *k13* sequence as a reference to detect point mutations in the gene. PCR and sequencing reactions were repeated when amplification did not occur or when the relevant portion of the sequencing chromatogram was unclear at any point.

### Analysis of *pfmdr1* copy number variation

Copy number variation in the *pfmdr1* gene was assessed using a *5-plex Rotor* Gene Q (Qiagen, Valencia, USA). The real-time PCR method was described previously [16, 19]. Briefly, each 10 µL PCR reaction contained 5 µL of 2x QuantiTect Multiplex PCR NoROX Master Mix (containing HotStarTaq DNA Polymerase and QuantiTect Multiplex PCR Buffer), 0.4 µM of each forward and reverse primer for both the *pfmdr1* gene and the *Pfβ*-*tubulin* gene, 0.2 µM of each probe, and 2.5 µL of template DNA. In each run of real-time PCR, 3D7 and H26 clones were used as calibration controls with single and multiple copies of *pfmdr1* gene, respectively, and negative control containing no template DNA was also included. Test samples were assayed in triplicate. The temperature profiles for real-time PCR were pre-denaturation at 95 °C for 15 min followed by 45 cycles of 94 °C for 60 s and 60 °C for 60 s. Fluorescence data were expressed as normalized reporter signal. The detection threshold was set above the baseline value for fluorescence of the first 15 cycles. The threshold cycle (Ct) is when the increase in reporter signal first rises above this threshold. Results were analysed by a comparative ∆∆Ct method where ∆∆Ct **=** **(**Ct of *Pfmdr1* gene−Ct of *P fβ*-*tubulin*) of sample- (Ct of *Pfmdr1* gene−Ct of *Pf β*-*tubulin*) of Pf 3D7 calibrator. The *pfmdr1* copy number was calculated as 2^∆∆Ct^. Samples with copy number between 1.45 and 1.6, and samples with a spread of ∆∆Ct among the three triplicates of more than 1 were repeated and the second result used.

### Statistical analysis

Qualitative variables were presented as proportions with confidence intervals calculated by Wilson’s method and comparison of proportions undertaken by the Chi squared test. Quantitative variables were expressed as median (IQR).

## Results

A total of 250 samples (76 from Ann, 111 form Homemalin, 38 from Myit Kyi Nar, 18 from Kyauk Me and seven from Phruso) were obtained for molecular analysis of K13 mutation and *pfmdr1* copy number variation (Additional file 1). Median parasitaemia was 9047 parasite/µl. *K13* sequencing and *pfmdr1* copy number assessment were both successful in 176 samples; samples in which either or both markers could not be reliably assessed were spread across the five sites.

### *K13* sequence

*K13* sequence was successfully obtained in 206 samples. Seventy-six samples (36.9 %) had non-synonymous mutations (19 different mutations, of which six are previously unreported) (Table 1). There was no sample with multiple *k13* mutations. Sixty-nine samples had non-synonymous mutations in the propeller region; 53 (76.8 %) of these had one of the mutations associated with artemisinin resistance according to the WHO definition of artemisinin resistance [20]: these were P441L, F446I, G449A, P574L, R561H, and A675 V.

**Table 1:** Numbers of non-synonymous mutations in *Plasmodium falciparum* K13 gene corresponding to amino acids 210–726 in different sites in Myanmar

	Ann	Homemalin	Myit Kyi Nar	Kyauk Me	Phruso	Total
Total n	64	91	32	13	6	206
Wild type	61^**^	59^**^	6	0	4	126
Non-propeller mutations						
R254L^*^			1			1
R255K		2				2
E294G^*^		1				1
I352T		2				2
R411K^*^		1				1
Total	0	6	1	0	0	7
%	0	6.6	3.2	0	0	3.4
(95 % CI)	(0.0–5.7)	(3.0–13.6)	(0.56–15.8)	(0–22.8)	(0–39.0)	(1.7–6.8)
Propeller mutations						
P441L			1			1
P443S		3				3
F446I		13	18	1		32
G449A	1				1	2
S485N					1	1
Y511H^*^			1			1
A529T^*^		1				1
N554L^*^	1					1
P574L	1		2	12		15
R561H		1				1
R575K		1				1
A675V		2				2
A676D		4	3			7
H719N		1				1
Total	3	26	25	13	2	69
%	4.7	28.6	78.1	100	33.3	33.5
(95 % CI)	(1.6–12.9)	(20.3–38.6)	(61.2–89.0)	(77.2–100)	(9.7–70.0)	(27.4–40.2)

* Newly identified mutations from this study

** Includes single samples with previously unreported synonymous mutations in Ann (E424E) and Homemalin (D343D)

**Fig. 1:**
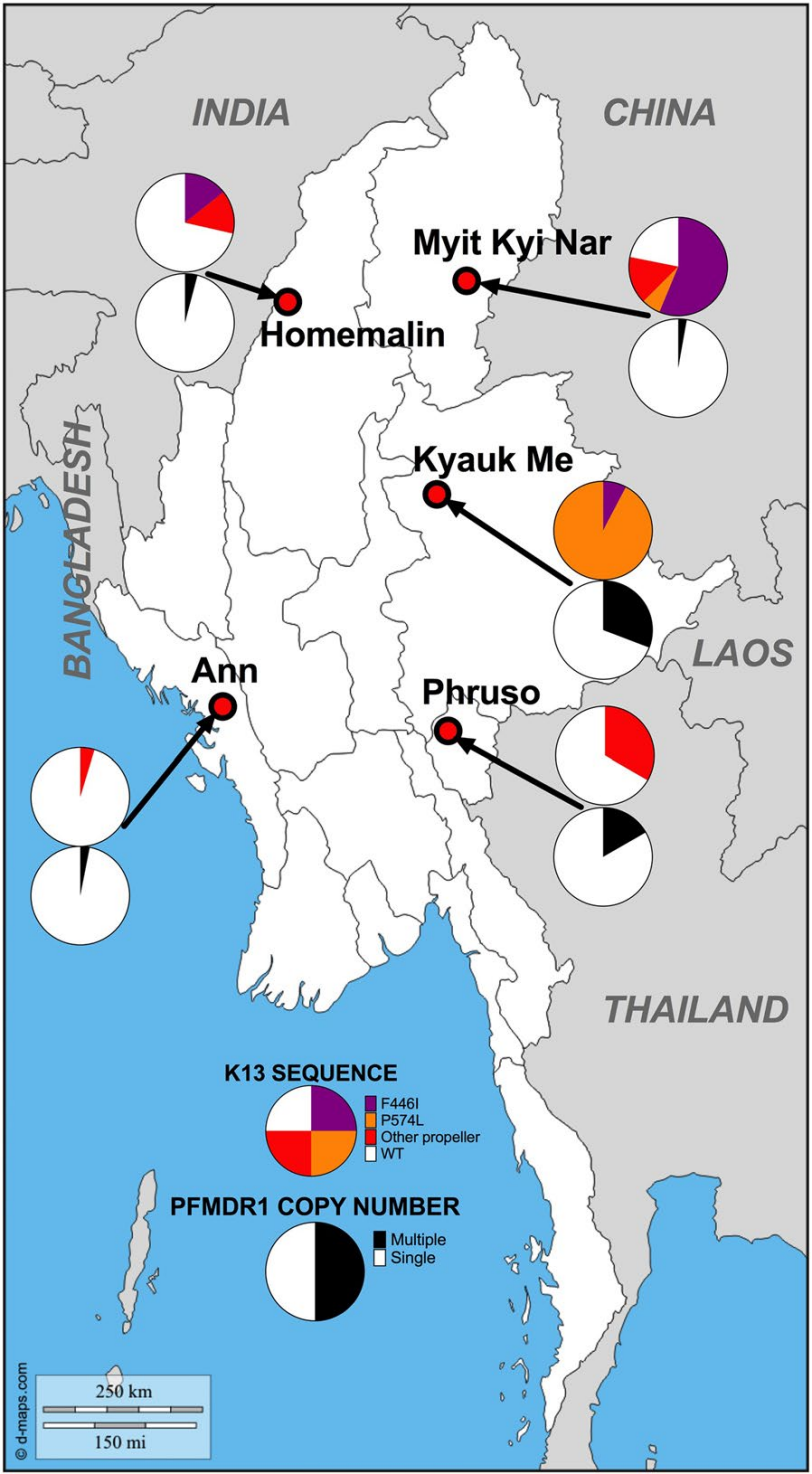
The prevalence of mutations in the propeller region of *Plasmodium falciparum* *k13* gene and *Pfmdr1* amplification in different sites in Myanmar

The overall prevalence of *k13* propeller mutations, and the most common mutation in each site differed among sites (Table 1; Fig. 1). Propeller mutation was less common in samples from sites in western border regions (Ann and Homemalin, 29 of 155 isolates) compared to samples from the other three sites (40 of 51 isolates; p < 0.0001). F446I was the most common mutation in Myit Kyi Nar and Homemalin while P574L was most common in Kyauk Me.

Five different non-synonymous mutations in the non-propeller (‘stem’) region of *k13* (corresponding to amino acids 210–440) were found in Homemalin or Myit Kyi Nar; three of these were previously unreported. Two infections with previously unreported synonymous mutations (one with E424E in Ann and one with D343D in Homemalin) were also detected.

### *pfmdr1* copy number variation

A total of 218 samples were successfully analysed for copy number variation in the *pfmdr1* gene. Using a copy number threshold of 1.5 to define multiple copies, only 12/218 (5.5 %) of samples had multiple copies (Table 2; Fig. 1). The overall median copy number across all five study sites was 1.03 (IQR = 0.86–1.2). The maximum copy number was 2.87 (in Homemalin). The proportion of samples having multiple copies was highest in Kyauk Me (four out of 13 samples). Only seven of 176 samples (4.0 %) with data for both genes had both *k13* propeller mutations and multiple copies of *pfmdr1*.

**Table 2:** *pfmdr1* copy number results by site

	Single	Multiple	% multiple	(95 % CI)
Ann	63	2	3.1	(0.8–10.6)
Homemalin	94	4	4.1	(1.6–10.0)
Myit Kyi Nar	35	1	2.8	(0.5–14.2)
Kyauk Me	9	4	30.8	(12.7–57.6)
Phruso	5	1	16.7	(3.0–56.4)
Total	206	12	5.5	(3.2–9.4)

### Day-3 parasitaemia after artemether-lumefantrine therapy

Blood films from 36 patients (26 in Homemalin and ten in Kyauk Me) taken on day 3 following completion of a directly observed regimen of artemether-lumefantrine therapy were obtained. Only one patient from Homemalin had a positive blood film at this time (parasite count: 50 parasites/µl). Molecular testing showed wild-type *k13* and single copy *pfmdr1.* Among the other 35 samples with negative day-3 parasitaemia, 19 were wild-type *k13*, nine had propeller mutations, two had non-propeller mutations and five could not be sequenced.

## Discussion

### *K13* assessment

This study adds to the knowledge of anti-malarial resistance in Myanmar. Approximately one-third of isolates had *k13* propeller mutations, with around three-quarters of these being mutations known to be associated with artemisinin resistance. Of the five locations surveyed, only Ann in Rhakhine State had a k13 propeller mutation proportion of less than 10 %. This finding is consistent with previous clinical and molecular data indicating that artemisinin resistance was not present in western Myanmar [9, 21], or in neighbouring Bangladesh [22]; the data also fit with lower levels of transmission (broadly found in the sites outside the western border regions) being associated with the most resistant parasites [23]. The high rate of mutation in Myit Kyi Nar (Kachin State) is consistent with data from neighbouring provinces of China where the F446I mutation is also the predominant type of *k13* mutation and associated with slow parasite clearance [12, 24] and reduced in vitro susceptibility [24]. F446I was also the most common mutation in Homemalin, near the northwest border with India, where it was found in 13 of 91 isolates (14.3 %). The P574L mutation was also present in three sites, consistent with its identification in seven provinces [18, 25]; it has been shown to be associated with a relatively long parasite clearance half-life [25].

Most *k13* mutations previously associated with slow parasite clearance (in Cambodia, Thailand and Vietnam) were not found in this study. This may reflect different levels of drug pressure and different population histories among study sites, although it is not possible to relate the mutations observed in different locations in Myanmar with particular drug usage histories, since these are largely unknown. The study also documents the presence at low frequency of a number of mutations in the ‘stem’ region of the *k13* protein outside the propeller region. Although clearly less common than propeller mutations, mutations in this highly conserved region may have functional consequences [26]. Further studies of *k13* mutation in conjunction with parasite clearance assessment or in vitro studies [27] should help to determine the relationship between this expanding list of observed mutations and phenotypic artemisinin resistance.

### *pfmdr1* copy number variation

In Southeast Asia and in laboratory studies, amplification of the *pfmdr1* gene has been shown to be associated with decreased in vitro sensitivity to both mefloquine and lumefantrine [16, 28] and it is associated with mefloquine-artesunate failure in Cambodia [29, 30], presumably in concert with propeller mutations in *k13* (present in high proportions in Cambodia for more than a decade). Recrudescences after the six-dose artemether-lumefantrine regimen have not been firmly linked with *pfmdr1* amplification [30], although relatively few artemether-lumefantrine efficacy data are available from areas with artemisinin resistance.

Prior to this study, few surveys had been undertaken examining copy number variation in the *pfmdr1* gene in Myanmar [31], although considerable data have been obtained from areas along the Thai-Myanmar [5, 32, 33] and China-Myanmar border areas [34, 35]. These studies have generally shown a prevalence of multiple copies of *pfmdr1* gene of less than 10 %. In the current study the overall median copy number was 1.03 (IQR = 0.86–1.2) and the proportion of samples with multiple copy numbers (more than 1.5) was less than 10 % in three out of five studied sites (Ann, Homemalin and Myit Kyi Nar).

This generally low rate of isolates with multiple *pfmdr1* copies across the country reflects the fact that although ACT, artesunate-mefloquine and artemether-lumefantrine, have been policy for treatment of *P. falciparum* malaria since 2005, it has been challenging to deploy them particularly in remote regions. Parasites carrying multiple copies of *pfmdr1* also have lower fitness in the absence of drug pressure [36]. The higher rates of multiple copies found in eastern areas (Kyauk Me and Phruso) may reflect the relatively small sample numbers (13 and six from Kyauk Me and Phruso, respectively). Nevertheless, the findings raise the suspicion of possible mefloqine resistance in these sites, perhaps related to population migration in and out of areas where relevant ACT are used. Molecular studies with larger sample size combined with clinical efficacy studies are needed to determine more accurately the drug resistance profile in these areas.

### Day-3 parasitaemia after artemether-lumefantrine therapy

Among 36 patients assessed in Homemalin and Kyauk Me for day-3 parasitaemia after completion of artemether-lumefantrine therapy, all patients cleared parasite on day 3 except one from Homemalin. This is quite surprising given the overall proportion of isolates with *k13* propeller mutations in these regions (28.6 % in Homemalin and 100 % in Kyauk Me), and the day-3 positivity rate appears lower than the previous clinical study done in Shwe Kyin in Lower Myanmar (9 %) [14]. Different starting parasitaemias might explain this discrepancy (minimum count was only 250 parasite/µl in the current study and median parasitaemia was consequently substantially lower). Another possibility is that the quantitative aspects of the relationship between *k13* propeller mutation and day-3 positivity may differ according to the specific *k13* mutation. Independent studies at the Myanmar-China border indicate that the proportion of day 3-positive patients after artemisinin derivatives is around 5–20 % but the proportion of isolates with *k13* propeller mutations (mostly F446I) was around 40–60 % [12, 24]. This differs from the situation in the lower Mekong region where the proportion of day 3-positive patients broadly matches that of *k13* propeller mutants [14]. Hence, certain mutations might have relatively mild phenotypes compared to better-characterized *k13* mutants, such as C580Y. A number of host variables (including immunity, pharmacology and genetics) might also be contributing factors. Further, the relatively small number of patients assessed for day-3 parasitaemia limits the precision of the estimate of parasite clearance time for artemether-lumefantrine therapy.

## Conclusions

This work describes two molecular markers of anti-malarial resistance, *k13* sequence and *pfmdr1* copy number, and hence enhances knowledge of the distribution of drug-resistant falciparum malaria in five regions of Myanmar. *K13* mutations are widespread, although with lower prevalence in western border regions. *Pfmdr1* copy number is generally single. The ACT currently used in Myanmar, including DHA-piperaquine [37], are likely to be efficacious at present, although regular monitoring through therapeutic efficacy studies will be important if development of widespread resistance to ACT is to be prevented.

## Additional file

10.1186/s12936-016-1147-3 K13 sequence, *pfmdr1* copy number and parasitaemia for all 250 samples.
