# Erratum to: K13 mutations and *pfmdr1* copy number variation in *Plasmodium falciparum* malaria in Myanmar

**DOI:** 10.1186/s12936-016-1346-y

**Published:** 2016-05-27

**Authors:** Aye A. Win, Mallika Imwong, Myat P. Kyaw, Charles J. Woodrow, Kesinee Chotivanich, Borimas Hanboonkunupakarn, Sasithon Pukrittayakamee

**Affiliations:** Department of Medicine, Institute of Medicine 1, Yangon, Myanmar; Department of Molecular Tropical Medicine and Genetics, Mahidol University, Bangkok, Thailand; Mahidol Oxford Tropical Medicine Research Unit, Bangkok, Thailand; Department of Medical Research (Lower Myanmar), Yangon, Myanmar; Nuffield Department of Clinical Medicine, Centre for Tropical Medicine & Global Health, University of Oxford, Oxford, UK; Faculty of Tropical Medicine, Mahidol University, Bangkok, Thailand

## Erratum to: Malar J (2016) 15:110 DOI 10.1186/s12936-016-1147-3

After publication of the original article [1], it was found that the affiliations of the first author were incorrectly stated. Dr. Aye A. Win is affiliated to both Department of Medicine, Institute of Medicine 1, Yangon, Myanmar, and Faculty of Tropical Medicine, Mahidol University, Bangkok, Thailand, which is reflected in this erratum.

## References

[1] Win AA, Imwong M, Kyaw MP, Woodrow CJ, Chotivanich K, Hanboonkunupakarn B, Pukrittayakamee S (2016). K13 mutations and *pfmdr1* copy number variation in *Plasmodium falciparum* malaria in Myanmar. Malar J.

